# MiRNA-7 Replacement Effect on Proliferation and Tarceva-Sensitivity in U373-MG Cell Line

**DOI:** 10.31557/APJCP.2020.21.6.1747

**Published:** 2020-06

**Authors:** Vahab Alamdari-Palangi, Zahra Karami, Hadi Karami, Maryam Baazm

**Affiliations:** 1 *Molecular and Medicine Research Center, Arak University of Medical Sciences, Arak, Iran. *; 2 *Department of Molecular Medicine and Biotechnology, Arak University of Medical Sciences, Arak, Iran. *; 3 *Department of Oral Medicine, Dental Research Center, Faculty of Dentistry, Hamadan University of Medical Sciences, Hamadan, Iran. *; 4 *Traditional and Complementary Medicine Research Center, Arak University of Medical Sciences, Arak, Iran. *; 5 *Department of Anatomy, Faculty of Medicine, Arak University of Medical Sciences, Arak, Iran. *

**Keywords:** Apoptosis, EGFR, Erlotinib, Glioblastoma, MiRNA-7

## Abstract

**Background:**

Deregulation of the EGFR signaling pathway activity has been shown to can be effective in resistance to EGFR-TKIs, such as Tarceva (erlotinib), in glioblastoma cells. In addition, reports have shown that the reduction of miRNA-7 expression levels is associated with an increase in the expression of EGFR. Here, we evaluated the effect of miRNA-7 on EGFR expression and sensitivity of the U373-MG glioblastoma to erlotinib.

**Methods:**

The effect of miRNA-7 on EGFR expression was examined using RT-qPCR and western blotting. Trypan blue and MTT assays were performed to explore the effect of treatments on cell growth and survival, respectively. The combination index analysis was used to evaluate the interaction between drugs. Apoptosis was measured by ELISA cell death assay.

**Results:**

We showed that miRNA-7 markedly inhibited the expression of EGFR and decreased the growth of glioblastoma cells, relative to blank control and negative control miRNA (p < 0.05). Introduction of miRNA-7 synergistically increased the sensitivity of the U373-MG cells to erlotinib. Results of apoptosis assay demonstrated that miRNA-7 can trigger apoptosis and enhance the erlotinib-mediated apoptosis.

**Conclusions:**

Our results show that miRNA-7 plays a critical role in the growth, survival and sensitivity of the U373-MG cells to erlotinib by targeting EGFR. Thus, miRNA-7 replacement therapy can become an effective therapeutic procedure in glioblastoma.

## Introduction

Glioblastoma multiforme (GBM) or grade IV astrocytoma is the most frequent brain tumor (Karsy et al., 2012; Garcia-Claver et al., 2013). Despite advances in surgical techniques, chemotherapy and radiotherapy, the median survival rate remains less than one year (Novakova et al., 2009; Li et al., 2013). Thus, development of new therapeutic strategies and tools is crucial for improving this situation. 

The epidermal growth factor receptor (EGFR) also known as ERBB1/HER1, is a member of the ERB family of receptor tyrosine kinases (RTKs) which is over-expressed in different types of malignant cells (Singh and Jadhav, 2018; Yamaoka et al., 2018). Over-expression of EGFR was found in more than 60% of primary GBM cases and is linked to the proliferation, angiogenesis and invasiveness of glioma cells (Koshkin et al., 2013; Li et al., 2013). EGFR activation triggers two primary intracellular signaling pathways, such as Ras/Raf/MAPK and the PI3K/Akt pathways, which enhances survival proliferation, migration and apoptosis resistance of certain tumor cells (Liu et al., 2018b; Sigismund et al., 2018; Singh and Jadhav, 2018). Therefore, the EGFR has considered as a major therapeutic target in cancer. Erlotinib (Tarceva), a small-molecule inhibitor that acts on the EGFR, is used as a second line treatment for glioblastoma therapy. While erlotinib have proven to be effective in treating other types of malignancies, it has produced poor results in glioblastoma patients (Garcia-Claver et al., 2013; Zahonero and Sanchez-Gomez, 2014). However, the exact mechanisms of the limited benefits of this agent had remained unclear.

MicroRNAs (miRNAs) are a class of small, noncoding transcripts and approximately 18-25 nucleotides, which bind to 3’-UTR of mRNAs to regulate gene expression, either via transcript degradation or translational inhibition (Jiang et al., 2018; Amri et al., 2019; Braicu et al., 2019). MiRNAs have been shown to affect multiple biological processes, such as cell growth, differentiation, angiogenesis, and apoptosis. Alterations in miRNA expression are a general feature in many human tumor cells, including glioblastoma, and they have been classified into oncogenic miRNAs and tumor suppressive miRNAs (Karpel-Massler et al., 2009; Ding et al., 2017; Gulluoglu et al., 2018; Liu et al., 2018a; Cui et al., 2019). For example, miRNA-641 expression is reduced in glioblastoma cells relative to normal brain tissue, causing elevated expression of MAPKAP1 and PIK3R3, increased tumorigenesis (Hinske et al., 2017). In contrast, miRNA-23a is over-expressed in glioblastoma cells, leading to repression of the homeobox D10 (HOXD10), resulting in profound tumor invasion (Yachi et al., 2018). Thus, miRNAs may serve as a target for effective glioblastomas therapy.

MiRNA-7 is a tumor suppressor that down-regulates in glioblastoma cells. It has been shown that reduced expression levels of miRNA-7 correlates with high levels of EGFR and upstream genes involved in Akt pathway (such as IRS-1 and IRS-2), leading to inhibition of migration, invasion and proliferation of glioblastoma cells (Kefas et al., 2008; Karsy et al., 2012; Babae et al., 2014). We hypothesized that miRNA-7 would able to sensitize glioblastoma cells to EGFR tyrosine kinase inhibitors (EGFR-TKIs) via the inhibition of EGFR. Therefore, we investigated the combination effect of miRNA-7 and erlotinib on growth, apoptosis and survival of glioblastoma cells. 

## Materials and Methods

*Cell culture*

The human U373-MG glioblastoma cells were purchased from Pasteur Institute (Tehran, Iran). Cells were cultured in RPMI-1640 medium (Sigma-Aldrich, St. Louis, MO, USA) supplemented with10% fetal bovine serum (FBS) (Sigma- Aldrich), 1% antibiotics (100 IU/ml Penicillin and 100 µg/ml streptomycin, respectively) (Sigma-Aldrich), 2 mM glutamine and 1% sodium pyruvate. Cultures were incubated at 37°C in an atmosphere containing 5% CO_2_ and used in the exponentially phase in all experiments.

*MiRNA transfection*

The miRNA-7 mimic and negative control (NC) miRNA lacking significant homology to all known human sequences were bought from Dharmacon (Lafayette, CO, USA). The sense strand sequences of miRNAs were: 

5’-UGGAAGACUAGUGAUUUUGUUGUU-3’, 

for miRNA-7 mimic and, 

5’-UUCUUCGAACGUGUCACGUTT-3’, for NC miRNA. 

MiRNA transfection (at a final concentration of 50 nM in all experiments) was performed using Lipofectamine™ 2000 transfection reagent (Invitrogen, Carlsbad, CA) according to the manufacturer’s protocol. 24 and 48 h after transfection, real-time quantitative PCR (RT-qPCR) was used to confirm the effect of miRNAs on expression levels of EGFR.

*RT-qPCR*

Total RNA was isolated from cancer cells by using YTzol reagent (Yekta Tajhiz, Tehran, Iran). Then, cDNA synthesis was performed using 1 µg of RNA and a Reverse Transcription System (Bio-Rad Laboratories, Hercules, CA, USA), according to the manufacturers protocol. The primers for EGFR were 5’-CCTGGTCTGGAAGTACGCAG-3’ and 5’- CGATGGACGGGATCTTAGGC-3’, and for β-actin, 5’-TCCCTGGAGAAGAGCTACG-3’ and 5’-GTAGTTTCGTGGATGCCACA-3’. RT-PCR amplification of cDNA was performed in a final volume of 20 µl, containing 12 µl of SYBR green reagent (Takara Bio, Otsu, Shiga, Japan), 0.2 µM of the primers, 1 µl of template cDNA and 6 µl of nuclease-free distilled water. RT-qPCR was performed in the LightCycler 96 System (Roche Diagnostics GmbH, Mannhein, Germany) by the blow procedure (95°C 5 min; 95°C 5 s, 60°C 25 s, 72 °C 25 s, 40 cycles). The relative EGFR expression level was determined with the 2 - (∆∆Ct) method and β-actin as the reference gene (Karami et al., 2014; Pirayesh Islamian et al., 2016; Azimi et al., 2018).

*Western blot analysis*

After exposure to miRNA for the indicated periods, the cells were washed twice with phosphate-buffered saline (PBS) and lysed in extraction buffer (1% SDS, 150 mM NaCl, 50 mM Tris-HCl, 1% Triton X-100, pH 7.4 and 1 mM EDTA, pH 8) containing protease inhibitor cocktail (Roche Diagnostics GmbH) at 4°C for 30 min. The cell suspensions were centrifuged at 12,000 g for 20 min at 4^o^C. The protein concentrations were quantified using Bradford reagent (Sigma-Aldrich). Identical amounts of each protein sample (fifty micrograms) were resolved in 8–10% SDS-polyacrylamide gels, and transferred to polyvinylidine diflouride membranes (PVDF) membrane (GE Healthcare, Amersham, Buckinghamshire, UK). After blocking the non-specific sites with blocking buffer (5% fat-free milk and 0.05% Tween-20 in PBS), the membranes were incubated overnight at 4oC with primary monoclonal antibodies against EGFR (1:700, Abcam, Cambridge, MA, UK) and β-actin (1:1,000, Abcam). The membranes were washed extensively, incubated with horseradish peroxidase (HRP)-linked goat anti-mouse secondary antibody (1:5,000, Abcam) for 2 h, and developed using enhanced chemiluminescence detection system Kit (GE Healthcare) and X-ray films (Estman Kodak, Rochester, NY, USA). The densities of the protein bands were measured by means of ImageJ 1.62 software (National Institutes’ of Health, Bethesda, Maryland, USA).

*Cell growth assay*

The effect of treatments on cell growth was determined by the trypan blue exclusion assay. Briefly, the U373-MG cells (1 × 10^5^ cells/ well) were treated with miRNA-7 and erlotinib in 6-well plates and incubated for 1-5 days. At different time points after treatments, the cells were collected and then cell suspension mixed with equal volume of 0.4% trypan blue stain (Merck KGaA, Darmstadt, Germany). After 2 min of incubation, the number of unstained cells (viable cells) was quantified using an inverted microscope (Nikon Instrument Inc., Melville, NY, USA) and a hemocytometer. Next, the percentage of viable cells was quantified by dividing the number of viable cells in experiment group by the number of viable cells in control group and multiplying by 100. Moreover, cell viability in each time was considered as 100% for blank control groups.

*MTT assay*

The 3-(4, 5-Dimethylthiazol-2-yl)-2, 5-Diphenyltetrazolium Bromide (MTT) assay was performed to determine the cytotoxic effects of miRNA-7 and erlotinib, alone and in combination. The assay was divided into eight groups: NC miRNA, miRNA-7, erlotinib, NC miRNA + erlotinib, miRNA-7 + erlotinib, miRNA blank control, erlotinib blank control and combination blank control. Cells treated with only 1% DMSO, solvent of erlotinib, or lipofectamine were served as erlotinib or miRNA blank controls, respectively. Briefly, cells were seeded in 96-well plates at a density of 5 × 10^3^ cells per well and transfected with miRNA-7 or NC miRNA at 50 nM concentration. Six hours after transfection, the cells were exposed to erlotinib at a final concentration of 2.5, 5, 10, 20, 40, 80 and 160 µM, and continued to further incubate for 24 and 48 h. Next, 10 µl MTT solution (Sigma-Aldrich) (5 mg/ml) was added to each well. After 4 h of incubation at 37 oC, the supernatants were discarded and 150 µl of DMSO was added to the cells. Absorbance (A) at 490 nm was read using an ELISA plate reader (Awareness Technology, Palm City, FL, USA). The survival rate (SR) was determined according to the equation as follows: SR (%) = (A Experiment /A Control) × 100%. IC_50_ (the concentration that reduced 50% of survival rate) was calculated by GraphPad software (GraphPad Software Inc., San Diego, CA, USA). 

*Determination of combination index values*

To further examine whether the combination of miRNA-7 with erlotinib is synergistic, the combination index (CI) values were calculated with Chou-Talalay CI model (Chou and Talalay, 1984). The results of the nonlinear dose–response curves were obtained from the MTT assay were converted to Fraction affected (Fa; range 0-1; where Fa = 0 is 100% cell survival and Fa = 1 is 0% cell survival) and analyzed using CompuSyn program from Combosyn (Paramus, NJ, USA). Synergistic, additive, and antagonistic effects are defined by CI value <1, =1 or close to 1, or >1, respectively. 

*Apoptosis ELISA assay*

The cells were cultivated at a density of 4 × 10^ 4^ cells/well in 24-well plates and then exposed to miRNA-7, erlotinib (IC_50_ dose) and their combination, as described previously. After 24 and 48 h of incubation, cytoplasmic DNA-histone complexes released from the nucleus into the cytoplasm of apoptotic cells were measured using an ELISA cell death detection kit (Roche Diagnostics GmbH) according to the supplier’s recommendations. The assay is based on quantitative sandwich ELISA assay principle, with monoclonal antibodies direct against DNA and histone, respectively. Briefly, the cells were harvested by trypsinization and lysed in incubation buffer for 20 min. The cell lysate was then centrifuged at 200 g for 10 min and supernatant was collected. Next, 20 µl of the cell supernatant and 80 µl of immunoreagent were added to each well of streptavidin-coated plate and incubated for 3 h at room temperature. After washing, 100 µl of ABTS solution was added to each well and reactions were stopped with ABTS stop solution. Finally, the absorbance was determined with an ELISA plate reader (Awareness Technology, Palm City, FL, USA) at 405 nm (with a reference wavelength of 490 nm).

*Statistical analysis*

Data are presented as mean ± standard deviation (SD). ANOVA followed by Bonferroni’s was used to evaluate statistical differences between groups, and values of p less than 0.05 were considered significant. All data were analyzed using GraphPad Prism statistical software.

## Results

*MiRNA-7 suppressed the expression of EGFR mRNA in U373-MG cells*

**Figure 1:**
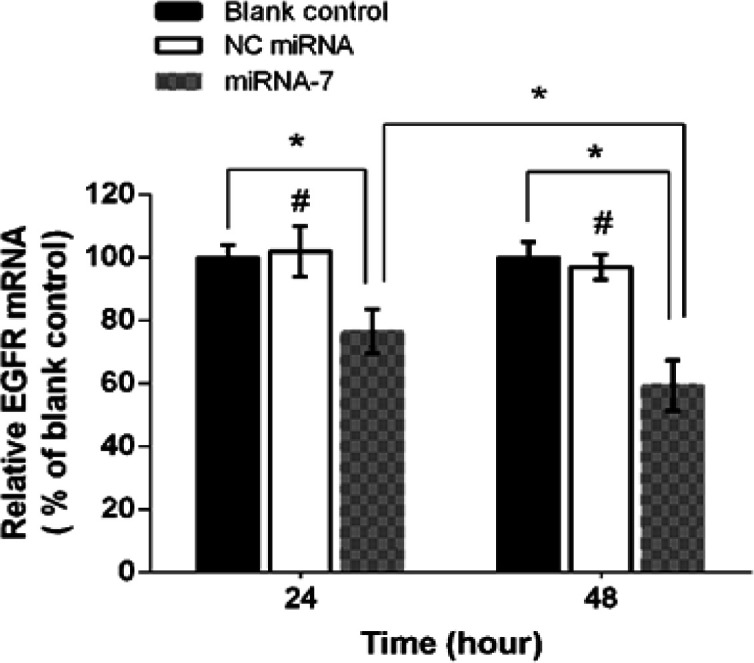
RT-qPCR Analyses of EGFR mRNA in U373-MG Cells. To measure the expression of EGFR in glioblastoma cells, the U373-MG cells were transfected with negative control (NC) miRNA and miRNA-7 for 24 and 48 h. Relative EGFR mRNA expression was quantified by RT-qPCR using 2 ^- (∆∆Ct)^ method and β-actin as an internal control. Data are presented as mean ± SD of three independent experiments. ^*^p < 0.05; ^#^*p* > 0.05 versus blank control

To analyzed whether miRNA-7 could influence the expression of EGFR in U373-MG cells, the cells were transfected for 24 and 48 hours with 50 nM NC miRNA and miRNA-7. Subsequently, the relative amount of EGFR mRNA was measured using real time PCR analysis. Results demonstrated that miRNA-7 transfection led to a time-dependent suppression of EGFR mRNA (Figure 1). After 24 and 48 h treatment with miRNA-7, the levels of EGFR mRNA were significantly reduced to 76.70% and 59.45% respectively, in comparison with blank controls (set at 100%). Meanwhile, treatment with NC miRNA did not modify the EGFR mRNA expression levels (p > 0.05).

*MiRNA-7 caused down-regulation of EGFR protein*

**Figure 2:**
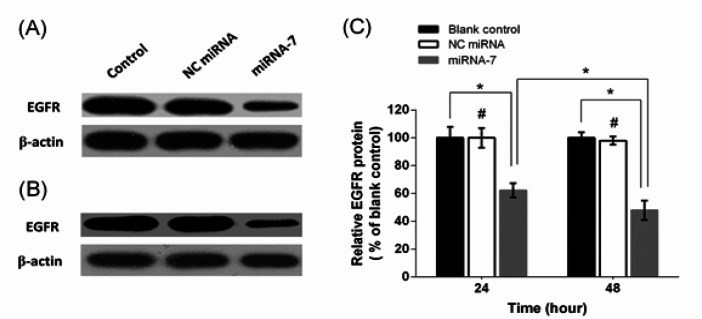
EGFR Protein Expression Levels in Glioblastoma Cells Transfected with miRNAs. Representative western blots of EGFR and β-actin proteins after 24 (A) and 48 (B). The effect of miRNA-7 on expression levels of EGFR was quantified using densitometry and normalized to the respective β-actin (C). The results are expressed as mean±SD of the results of three independent experiments. ^*^p < 0.05; ^#^*p* > 0.05 versus blank control

Western blotting was performed to assess the effect of miRNA-7 of EGFR protein expression. Results showed that miRNA-7 transfection significantly lowered the expression of EGFR protein to 62.30% and 47.95% after 24 and 48 h, respectively (p<0.05; Figure 2C). However, there was no distinct difference in EGFR protein expressions levels between NC miRNA and blank control group (p>0.05; Figures 2A, 2B, 2C).

*Inhibition of cell growth by miRNA-7*

**Figure 3:**
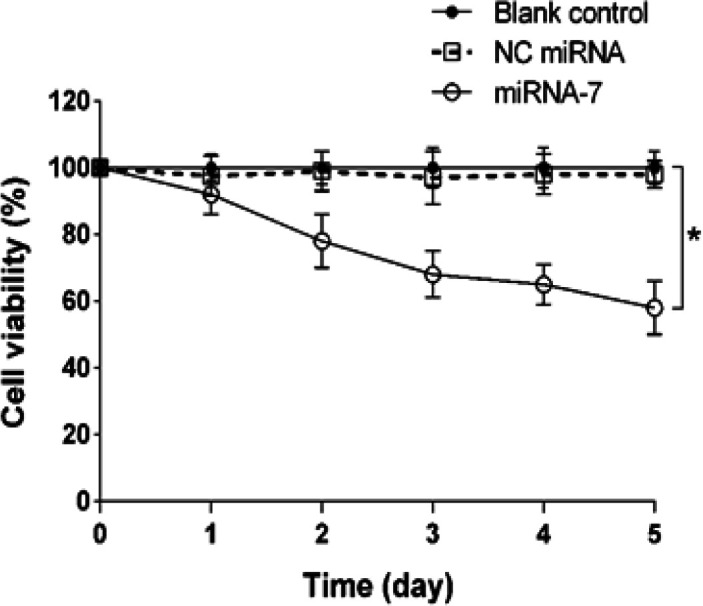
Growth Inhibition of U373-MG Cells Transfected with miRNA-7. The cells were transfected with negative control (NC) miRNA and miRNA-7 for 24-120 h. The cell growth rate was analyzed by trypan blue exclusion assay at the end of each period. The results are represented as mean ± SD (n=3). ^*^p < 0.05 versus blank control or NC miRNA

As over-expression of EGFR is associated with the growth of the glioblastoma cells; we therefore sought to test whether up-regulation of miRNA-7 could inhibit the cell growth. The U373-MG glioblastoma cells were transfected with miRNA-7 and NC miRNA and cell viability was then assessed every 24 h for 5 days. Results showed that compared with blank control group, miRNA-7 markedly inhibited the cell viability in a time dependent manner (p < 0.05; Figure 3). The inhibitory effect of miRNA-7 on cell viability was 92.33% at 24 h, 78.52% at 48 h, 68.20% at 72 h, 65.85% at 96 h and 58.94% at 120 h. In contrast, no significant changes in cell growth were observed between the NC miRNA transfected cells and the blank controls (p > 0.05; Figure 3).

*MiRNA-7 increased the cell toxicity of erlotinib in U373-MG glioblastoma cells*

**Figure 4:**
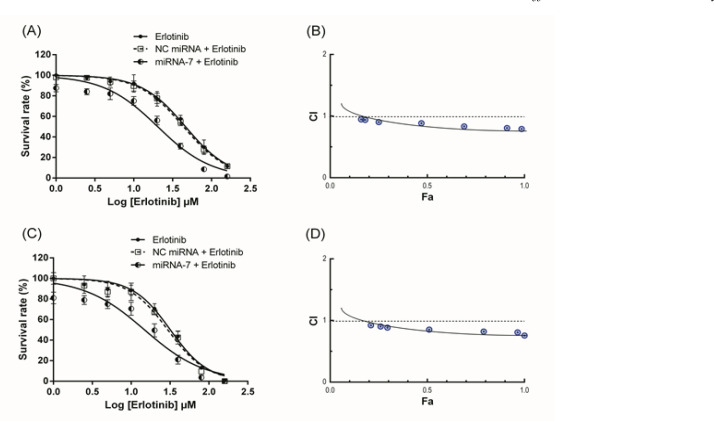
Synergistic Effect between miRNA-7 and Erlotinib in Human U373-MGCells. Twenty-four (A and B) and forty-eight (C and D) hours effect of miRNA-7 on the sensitivity of the glioblastoma cells to erlotinib were measured by MTT assay as described in the method section. The cell survival curves were plotted using GraphPad software. The results are expressed as mean ± SD of three independent experiments. Combination index (CI) versus fractional effect (Fa) was plotted using the Chou-Talalay method and CalcuSyn software. A horizontal dashed line shows CI of 1

Cytotoxicity of erlotinib and the effect of miRNA-7 on erlotinib-induced cytotoxicity were investigated in the U373-MG cells using MTT assay. Results of MTT assay revealed that treatment with erlotinib alone enhanced cell toxicity in a dose-dependent way (Figures 4A and 4C). Moreover, transfection of miRNA-7 for 24 and 48 h significantly lowered the cell survival rate to 87.11% and 81.64% respectively, relative to the blank control (p < 0.05). MiRNA-7 in combination with erlotinib further reduced the cell survival relative to miRNA-7 or erlotinib alone (p < 0.05). Surprisingly, miRNA-7 markedly reduced the IC_50_ values of erlotinib from 46.27 µM to 20.30 µM and 31.92 µM to 14.48 µM after 24 and 48 h of transfection, respectively (Table. 1). Meanwhile, the combination treatment with NC miRNA and erlotinib did not differ from NC miRNA treatment alone or erlotinib monotherapy (p > 0.05; Figure 4 and Table. 1).

*MiRNA-7 in combination with erlotinib inhibits the cell survival of glioblastoma cells in a synergistic manner*

The effect of combining the therapies was determined by the CI theorem of Chou–Talalay using CompuSyn software. Our results demonstrated that, the effect of combination treatments on cells survival was synergistic and the CI values were less than 1 in all concentrations of erlotinib combined with miRNA-7 (50 nM) (Figures 4B and 4D). CI–Fa plots further indicated that the strongest synergism effects of 24 h (CI=0.79) and 48 h (CI=0.75) of treatments were occurred at 160 µM of erlotinib with Fa levels of 0.98 and 1, respectively (Table 2).

*Up-regulation of miRNA-7 enhanced erlotinib-induced apoptosis*

**Figure 5:**
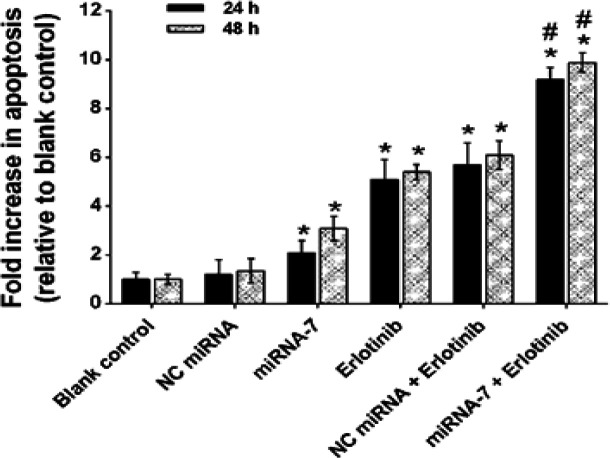
Effect of miRNA-7 on Erlotinib-Mediated Apoptosis in U373-MG Cells. The cells were treated with miRNA-7 (50 nM), negative control (NC) miRNA (50 nM) and erlotinib (IC50 doses of 24 and 48 h), alone and in combination. After 24 and 48 h, apoptosis was measured by cell death ELISA assay. The data are expressed as mean ± SD (n=3). *p < 0.05 relative to blank control; #p < 0.05 relative to miRNA-7 or erlotinib alone

To confirm whether the synergistic effects observed in MTT assay were linked to the increase in the extent of apoptosis, the ELISA cell death assay was performed. As shown in Figure 5, transfection of miRNA-7 alone for 24 h augmented apoptosis by 2.17 fold, whereas exposure of the cells with erlotinib alone caused 5.36 fold increases in the apoptosis (p < 0.05, compared with the corresponding controls). In contrast, the combination of miRNA-7 with erlotinib further increased apoptosis to 9.55 fold (p < 0.05, compared to the monotreatment). In the cells treated with the miRNA-7 and erlotinib for 48 h, the apoptosis was also enhanced by 3.20 and 5.48 fold, respectively. Moreover, miRNA-7 in combination with erlotinib significantly increased the level of apoptosis by 9.90 fold in this time (p < 0.05, compared to monotherapy). NC miRNA alone or in combination with erlotinib displayed no enhanced rate of cellular apoptosis compared to the miRNA-7 or erlotinib, respectively (p > 0.05; Figure 5). These results are consistent with the hypothesis that the sensitization effect of miRNA-7 is contributed to the induction of apoptosis.

**Table 1:** IC_50_ of Erlotinib Alone and in Combination with miRNAs in U373-MG Cells, after 24 and 48 h of Treatment

Treatment	IC_50_ (µM)	
	24 h	48 h
Erlotinib	46.27 ± 1.18	31.92 ± 2.20
NC miRNA and erlotinib	43.61 ± 1.10#	29.59 ± 0.80#
miRNA-7 and erlotinib	20.30 ± 1.25*	14.48 ± 1.45*

IC_50_ were determined by sigmoidal dose-response model using GraphPad software. Results expressed as the mean ± SD (n=3). *p < 0.05 versus corresponding erlotinib group; ^#^*p* > 0.05 relative to the corresponding erlotinib.

**Table 2:** CI Analysis of Combination Treatment in U373-MG Cells

Erlotinib concentration (µM)	24 h			48 h		
	Fa	CI	Combined effect	Fa	CI	Combined effect
2.5	0.16	0.94	S	0.21	0.92	S
5	0.18	0.93	S	0.26	0.9	S
10	0.25	0.90	S	0.29	0.87	S
20	0.47	0.88	S	0.51	0.85	S
40	0.69	0.83	S	0.79	0.82	S
80	0.91	0.80	S	0.96	0.80	S
160	0.98	0.79	S	1.00	0.75	S

The effects of miRNA-7 and erlotinib combination treatments were determined with the combination index (CI) analysis based upon the Chou-Talalay method. A CI values less than 1, equal to 1, or greater than 1 indicates synergistic (S), additive, or antagonistic interaction, respectively.

## Discussion

Glioblastoma is the most common subtype of malignant brain tumors, accounting for more than 50% of glioma. Despite abundant therapeutic efforts, only few patients are cured (Novakova et al., 2009; Karsy et al., 2012; Garcia-Claver et al., 2013; Li et al., 2013). Thus, new clinical investigations are urgently needed to substantially improve the prognosis of patients.

Erlotinib is a HER1/EGFR-specific reversible TKI that binds to the cytoplasmic ATP pocket domain of the receptor, blocking cell-cycle progression, inducing apoptosis and suppressing the growth of human tumor cell lines and xenografts models in mice (Raizer, 2005; Brandes et al., 2008; Taylor et al., 2012). Although data from in vitro studies evaluating erlotinib have been very promising; however, this drug has shown limited efficacy in glioblastoma patients due to the recurrent problem of resistance (Reardon and Wen, 2006; Taylor et al., 2012). Some of the potential mechanisms that could determine sensitivity to EGFR-TKIs include point mutations in the extracellular domain of EGFR, EGFR copy number, Akt activation, mutations in the tumor suppressor gene phosphatase and tensin homologue (PTEN), and activation of alternative signaling pathways (Raizer, 2005; Brandes et al., 2008; Taylor et al., 2012; Kalman et al., 2013). However, the precise molecular mechanisms underlying resistance in glioblastoma are not clearly identified. In the present study, we applied miRNA-7 to knockdown EGFR in the U373-MG glioblastoma cells, and evaluated the role of miRNA-7 in response of glioblastoma cells to erlotinib.

MiRNAs are highly conserved regulatory non-coding RNAs that play pivotal roles in many biological processes such as cell proliferation, differentiation, cell cycle control, and apoptosis (Novakova et al., 2009). Recent data demonstrated that dysregulation of miRNAs is an integral process of cancer development and progression. Moreover, abnormalities in miRNA expression are associated with the glioblastoma pathogenesis (Kefas et al., 2008; Li et al., 2013). MiRNA-7 is a tumor suppressor that inhibits the growth, survival, migration and reduces drug resistance in various cancer cell types through different mechanisms (Novakova et al., 2009; Karsy et al., 2012; Li et al., 2013). Here, we found that introduction of miRNA-7 alone, markedly inhibited the growth of U373-MG cells, supporting its critical role in the proliferation of lung cancer cells. Moreover, transfection of miRNA-7 decreased the cell survival rate and triggered apoptosis. These results further confirm that miRNA-7 can act as a tumor suppressor miRNA in glioblastoma cells. Previous studies have showed that miRNA-7 down-regulates the expression of EGFR and key modulators of the Akt pathway including IRS-1 and IRS-2 in glioblastoma, breast, cervical and lung cancer, which led to decreased cell survival and proliferation (Kefas et al., 2008; Webster et al., 2009; Kalinowski et al., 2014). Furthermore, miRNA-7 has been shown to target anti-apoptotic genes in lung and cervical cancer cells, where ectopic miRNA-7 expression enhanced cellular apoptosis (Li et al., 2013; Kalinowski et al., 2014). However, these observations are in agreement with our results and illustrate the important biological role of miRNA-7 in glioblastoma cells.

EGFR is a member of the RTKs which is over-expressed or hyperactivated in different types of human malignancies including glioblastoma (Koshkin et al., 2013; Li et al., 2013; Singh and Jadhav, 2018; Yamaoka et al., 2018). These aberrations trigger Ras/Raf/MAPK and the PI3K/Akt intracellular signaling pathways, which subsequently lead to enhanced proliferation, migration and impaired apoptosis of tumor cells (Liu et al., 2018b; Sigismund et al., 2018; Singh and Jadhav, 2018). In this study, we observed that exposure of the U373-MG cells to erlotinib reduced the cell survival rate and triggered significant apoptosis relative to the blank control. Introduction of miRNA-7 to glioblastoma cells markedly decreased EGFR expression levels and synergistically enhanced the cytotoxicity of erlotinib. Furthermore, miRNA-7 enhanced sensitivity of the tumor cells to erlotinib-mediated apoptosis. These findings proposes that up-regulation of miRNA-7 could render glioblastoma cells sensitive to EGFR-TKIs via blockage of EGFR. In agreemnt with our findings, Kefas et al., (2008) showed that miRNA-7 was down-regulated in glioblastoma tissue versus surrounding brain. The results of their study demonstrated that miRNA-7 directly suppresses EGFR and upstream modulators of the IGF-1R/Akt signaling pathway, IRS-1 and IRS-2 proteins.

Further studies have shown that miRNA-7 has the potential to enhance the sensitivity of the cancer cells to the various therapies. For example, Kalinowski et al., (2014) demonstrated that over-expression of miRNA-7 increased response to erlotinib in erlotinib-resistant head and neck cancer cells by inhibiting Akt activity. Other study also showed that lower levels of miRNA-7 are associated with the elevated levels of multidrug resistance-associated protein 1 (MRP1) expression and resistance to cisplatin in breast cancer cells. Moreover, it has been shown that enforced expression of miRNA-7 caused down-regulation of EGFR and enhanced sensitivity to radiotherapy in glioma cells (Lee et al., 2011). However, the results of these reports are similar to that found in our study and suggest that down-regulated miRNA-7 expression is related to EGFR over-expression and may be contribute to erlotinib-resistance of glioblastoma cells. 

In conclusion, we have showed that up-regulation of miRNA-7 is associated with growth inhibition of glioblastoma cells and enhancement of sensitivity to erlotinib. Moreover, we have provided evidence to confirm the role of miRNA-7 in glioblastoma erlotinib resistance, possibly in part via regulation of EGFR expression. Taken together, our results suggest that miRNA-7 may act as a key therapeutic target for glioblastoma. 
